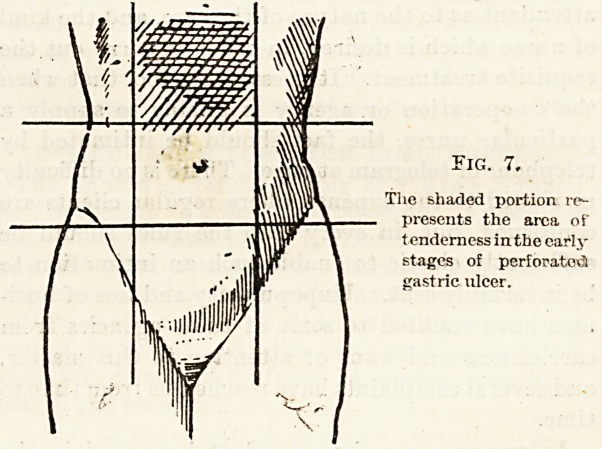# "The Hospital" Nursing Section

**Published:** 1906-04-28

**Authors:** 


### The Hospital.

### Nursing Section.

Contributions for "THE HOSPITAL," should be addressed to the EDITOR, "THE HOSPITAL" NURSING SECTION, 28 & 29 Southampton Street, Strand, London, W.C.

No. 1,022.—VOL. XL. SATURDAY, APRIL 28, 1906.

### Notes on News from the Nursing World.

### THE SAN FRANCISCO DISASTER.

THE awful disaster at San Francisco, of which the whole world is talking, must have taxed the resources of nurses on the spot to the utmost. We shall learn more fully later on how they rose to the occasion—an occasion unprecedented in history. But at the outset of the calamity nurses, as well as medical men, volunteered in numbers, and tended the sufferers injured by earthquake and fire in the Mechanics' Pavilion, so long as that structure was available as a temporary hospital. The last of the patients was removed elsewhere just before fire shot from the roof, the nurses refusing to leave until the police insisted. Elsewhere there were terrible scenes particularly at the Agnes State Hospital, near Santa Clara, which was entirely destroyed, a large proportion of the insane patients being killed, while many more were badly hurt. Here, too, both nurses and medical men remained at their posts and did their utmost both to succour the injured and to prevent the dangerous uninjured from making their escape.

### THE SETTLEMENTS IN SAN FRANCISCO.

BEFORE the disaster at San Francisco the staff attached to the Nurses' Settlement had been hoping to establish a small free hospital, as they had frequently experienced difficulty in persuading their patients to enter the large institutions, or in gaining the parents' permission to the removal of their children. Now, of course, all possibility of carrying out the scheme is at an end for the time, and it is not even known whether the nurses themselves have escaped with their lives, as the Settlement was naturally in the poorer parts of the city, which suffered the most from the shock. It is eight years since the pioneer Settlement was first started. The work is very varied in character, including not only nursing the sick by day or by night, but visiting four of the public schools, inspecting two kindergartens, reporting to the authorities cases of overcrowding or insanitary surroundings, and, in fact, acting as auxiliary health inspectors, the Board of Health giving them badges of office. When not actually engaged in nursing, the nurses instruct the mothers about their babies and the principles of hygiene, and teach the boys and girls at the clubs in the winter evenings, or in the summer out at the farm attached to the Settlement. In fact, as one of the nurses says, "Our life proves that hospital training for a district should include knowledge of farming, camp cooking, and dancing as well as care of the sick!"

### FAREWELLS TO MISS BROWNE.

ON Thursday, last week, a farewell dinner was given Miss Sidney Browne, R.R.C., by her colleagues in the Medical Department of the War Office. The entertainment was of a purely private character, and was followed on Saturday afternoon by a reception given at the Windsor Hotel by the members of Queen Alexandra's Imperial Military Nursing Service. This reception, also, was of a private character, but we are informed that the friends of Miss Browne came from far and near to take the opportunity of expressing their deep regret at Miss Browne's departure after her 26 years of service. Among the numerous guests present were Countess Roberts, Mrs. Keogh, wife of the Director-General, Mrs. St. John, Miss Monk, Miss Cave, Colonels Skinner, Harrison, and O'Keefe. During the afternoon many telegrams were received by Miss Browne bidding her farewell from those who were unable to attend the reception.

### THE ARMY NURSING BOARD.

WE understand that Miss Stewart, matron of St. Bartholomew's Hospital, has been appointed to the vacancy on the Nursing Board of Queen Alexandra's Imperial Military Nursing Service, caused by the resignation of Miss Monk. We scarcely think that she has been selected because, as it is suggested, she is, "the one London hospital matron who worked hard for Army nursing reform," for this is not correct. But she should bring to bear upon the performance of her new duties most valuable experience.

### POOR-LAW NURSES AND SUPERANNUATION.

WHEN the Superannuation Act of 1896 was passed every nurse was asked to decide whether she would come under it or be excluded from its benefits. Many nurses, thinking only of the deduction proposed to be made from their small salaries, chose the latter alternative. After the Act came into operation, every Poor-law officer subsequently appointed was regarded as coming under its provisions. This regulation had the effect of discouraging nurses from entering the Poor-law service, and eventually it was amended so as to permit all the opportunity of contracting out. In 1898, however, the Local Government Board decided that a nurse who had once contracted out of the Superannuation Act could not subsequently contract in again. This decision has since been modified in view of difficulties which have arisen, and the Local Government Board have now determined that if a nurse has contracted out of the

Act of 1896, she is bound by her decision in every subsequent appointment she may hold as nurse within the meaning of the Act of 1897; but that, if she be appointed to any other office than that of nurse, she will come under the operation of the Act of 1896. Thus, a nurse, on becoming a matron, will be allowed to come under the Superannuation Act, the recognised percentage will be deducted from her emoluments, and she will be eligible for a pension at the end of her service. But the serious point is that no part of her service as a nurse will be reckoned as a part of the service which qualifies for her pension. It is true that if during her service as nurse the officer has contributed no part of her salary towards the Superannuation Fund, this course is strictly legal. On the other hand, the years which are to be left out of account may have been the best of her life, and it is quite possible that she has frequently regretted her hasty decision to throw aside the advantages of the Superannuation Act. It seems to us that it would be better if nurses came under the provisions of the Act as a matter of course on entering the Poor-law service. They would find the small deduction—usually 2½ per cent.—from their emoluments a trifling loss. If they leave the service before qualifying for a pension they might, as in the Royal National Pension Fund for Nurses, receive back the premiums they have paid, but without interest. Or, if some still prefer the right of contracting out of the Act, let them be given the opportunity of reversing their decision if at any subsequent time they change their opinion. In that case their term of service for Superannuation purposes could be reckoned only from the time when they came under the provisions of the Act.

### DEWSBURY GUARDIANS AND THEIR NURSES.

The frequent vacancies in the nursing staff at Dewsbury Poor-law Infirmary have prompted a committee which has been considering the matter to recommend the apointment of a male attendant for the bathing of all "lock" cases; the appointment of four additional probationers in order to bring the staff up to the level of four charge nurses and fourteen probationers, the provision of additional sleeping accommodation for the staff, and the revision of the duties of the nurses. These appear to be excellent recommendations, though, obviously, much may depend upon the manner in which the last two are carried out. Certainly, the Dewsbury Guardians should not allow the bathing of "lock" cases by female nurses.

### THE IMPORTANCE OF AN OUTSIDE INTEREST.

We agree with a correspondent, who, in a letter headed "A Plea for Hobbies," insists that it is essential for a nurse to have some interest outside her work. Our correspondent urges that the interest should be absorbing, but we prefer her alternative way of putting it, that, at any rate, the interest should be sufficiently great to make the nurse forget her work during the time she is pursuing her hobby. On all grounds it is desirable that the attention of nurses should be diverted from their duties when they are not in the wards, and we welcome with much pleasure the growing tendency to form good libraries for their benefit in the institutions to which they are attached. There are other and more healthy recreations in summer, but reading is one in which it is exceptionally easy to participate at any time. Hobbies cannot always be pursued in off-duty hours.

### AN IRISH BISHOP ON SMALL SUBSCRIPTIONS.

The principal speaker at the annual meeting of the Clonmel Jubilee Nursing Association was the Bishop of Waterford and Lismore, who presided. Having remarked that the balance to the credit of the Association was £13 more than at the end of the previous year, he urged the importance of depending upon a very large number of small subscriptions rather than upon a very limited number of large subscriptions. He maintained, moreover, that the persons who can give the small subscriptions are most called upon to help the Society, because they derive advantages from it, rather than the rich, who can afford to employ nurses on their own account, or the poor who make use of the hospitals. This is an argument that might be made use of in other towns than Clonmel, and we are glad that the Bishop strongly emphasised the unwisdom of expecting a nursing association deriving its income from a few contributors to continue in a flourishing condition.

### MUSIC IN LEISURE HOURS.

At the last meeting of the Knaresborough Rural Council, exception was taken to the provision for the nurses of the Harrogate and Knaresborough Isolation Hospital of a piano. The Chairman, however, explained that the instrument had simply been transferred from the old hospital to the new building, adding that he did not think the nurses should be denied the pleasure of a little music in their leisure hours. We agree with him. Moreover, in the case of nurses in hospitals for infectious diseases, the need is rather greater than less, because in off-duty time the choice of recreation is exceptionally limited.

### A SOUND POSITION AT BURSLEM.

One of the satisfactory features of the report which was submitted at the annual meeting of the Burslem District Nursing Institution was that the nourishment fund had been sufficient for the demands, the nurses not having encountered the same amount of distress which had prevailed in the previous year. In connection with the Institution the Alcock Memorial Convalescent Fund is a source of considerable strength, no fewer than 16 girls having been sent away to a country home during the year. As there was a balance of nearly £10 on the year's working, the financial position must also be pronounced satisfactory, and we note with pleasure that by far the bulk of the income is derived from subscriptions.

### IMPERIAL MILITARY NURSING SERVICE.

We are officially informed that Miss L. W. Tulloh, R.R.C., matron in Queen Alexandra's Imperial Military Nursing Service, has been transferred to the Royal Infirmary, Dublin, from the Military Hospital, Canterbury. Miss A. Guthrie, sister, has been transferred to the Military Hospital, Harrismith, Orange River Colony, South Africa, from Middelburg, Cape Colony; Miss A. Nixon, sister, to the Royal Victoria Hospital, Netley, on her return from South Africa; Miss A. S. Bond, R.R.C., sister, to the Military Hospital, Canterbury, from the Royal Victoria Hospital, Netley; and Miss M. E. Harper, R.R.C., sister, to South Africa, from sick leave at home. Miss M. S. Ram, staff nurse, has been transferred to the Royal Military College, Sandhurst, from the Royal Herbert Hospital, Woolwich; and Miss C. A. Coats, sister, to the Queen Alexandra Military Hospital, Millbank, on appointment.

### THE NEW MATRON OF GRIMSBY HOSPITAL.

The election of the new matron of Grimsby and District Hospital took place on Monday, the successful applicant being Miss E Davies, matron of the Royal Cornwall Infirmary, Truro. The appointment is an excellent one. Miss Davies has enjoyed precisely the sort of experience which should fit her for her new work in the still rapidly rising Lincolnshire seaport. Trained at the Portsmouth Royal Hospital, she has since been in succession staff nurse and sister at a number of important institutions, including Bristol Royal Infirmary, where she was first sister and subsequently night superintendent and home sister. In July 1903 she was appointed matron of the Royal Cornwall Infirmary, and an account of the nursing at this institution under her auspices appeared in our columns on September 19 of that year. The conditions under which she will fulfil her duties as matron at Grimsby are not dissimilar, and she will certainly find that the encouragement given to her by the authorities at Truro is not withheld in her new sphere of labour. Miss Crichton leaves Grimsby on May 22, and Miss Davies succeeds her on the same day.

### FUNCTION AT MOUNT VERNON HOSPITAL.

On Tuesday last an "at home" and annual prize-giving to the nursing staff of the Mount Vernon Hospital for Consumption and Diseases of the Chest, Hampstead, took place. A number of the members of the Hospital Committee and nurses' friends were present. The Chairman, Mr. Henry Stedall, in presenting the prizes, expressed the very great pleasure and satisfaction it gave the Committee to receive such a good report of the result of the nurses' examinations, and the way in which they had done their work during the past year. Of the second-year nurses Nurse Kelly received the first prize, and Nurse Thompson the second for general efficiency; Nurse Pickering first and Nurse Muncey second for anatomy and physiology; and Nurse Robinson first, and Nurses Pickering and Muncey second for dispensing theory and practice. Of the first-year nurses, Nurse McNeill was awarded first and Nurse Caldeclough second for nursing lectures and general efficiency; Nurse Ricketts first and Nurse McNeill second for anatomy and physiology. The first prize in general efficiency in the second year is a green belt with silver clasp, inscribed, to be worn by the nurse as a badge of honour and distinction during the remainder of her stay in the hospital. In alluding to the year's work by the nurses, the matron, Miss Swain, stated that she had taken some of the nurses for three months at a time and given them instruction in housekeeping and general hospital management. She expressed herself well satisfied with their work. After the presentation the room was cleared and a most enjoyable musical evening was spent.

### EXAMINATION QUESTIONS AT LIVERPOOL.

At the usual examination of the nurses of Mill Road Infirmary, Liverpool, which has just been held, the first-year nurses were required to describe in writing the contents of the abdomen and to give a short account of the digestion; the second-year nurses to mention the various kinds of fractures, and to state how they would prepare for a fracture of the femur and stop the bleeding from a varicose vein in the leg; while the third-year nurses were asked by the examiner, Dr. Nathan Raw, to supply a full account of the aseptic system of surgery, and to describe a typical case of acute lobar pneumonia, mentioning the chief points in the nursing of the case and filling in a temperature, pulse, and respiration chart. Prizes were awarded respectively to Nurses Poste and Cannon, Nurses Bullock and Tunstall, and Nurses Mackintosh and Farnsworth.

### KING EDWARD'S FUND FOR IRISH NURSES.

The annual general meeting of "King Edward the Seventh's Coronation National Fund for Nurses" will take place in Dublin on Monday, May 14. The Countess of Aberdeen has become patroness of the Society, which at its last quarterly meeting made a grant of £12 to a nurse member in need of assistance. At the same meeting applications for membership from 14 nurses were considered and accepted.

### SHORT ITEMS.

We hear with pleasure that Miss Oxford's "Handbook of Nursing" has just been translated into French, the translator, Mlle. Chaptal, having adapted it for the use of the *Maison-Ecole d'infirmières privées*, of which she is a founder. The translation is published by M. Masson, of Paris.—Mr. Howard Morley has contributed £1,000 towards the current expenses of Queen Victoria's Jubilee Institute for Nurses.—New furniture for the whole of the nurses' and doctor's quarters in Tottenham Hospital has been presented by Mr. Harris Lebus, of Tottenham Hall, in recognition of the work which the institution is doing amongst the poor in the district.—The third annual meeting of the Rural Midwives Association will be held, by kind permission of Lady Esther Smith, at 3 Grosvenor Place, on Thursday, May 3, at three o'clock, Sir Michael Foster, K.C.B., in the chair.—Miss Weighall, R.R.C., has been re-appointed lady superintendent of Lady Roberts Hospital for Officers at Murray, Punjab, and she left England last week in the s.s. *Arabia* to take up her duties in India.—The Japanese Red Cross Society has decided to despatch a hospital ship, with a large staff of nurses, to San Francisco.

### The Nursing Outlook.

"From magnanimity, all fears above;
From nobler recompense, above applause,
Which owes to man's short outlook all its charm."

### THE SUPPLY OF NURSES TO THE PUBLIC.

The organisation of private nurses is firstly a matter of supply and demand. The private nurse who has few or no friends has to become associated with a co-operation or a supply agency in order to keep in regular employment. The profession and the public rely upon the supply centres, and have the right to expect that these shall be so organised as to secure the maximum co-operation between the employers and employed. No co-operation or agency can hope to maintain its business unless it secures full consideration for all who apply to it. It is essential that exact attention be given to the instructions which may be received from the medical attendant as to the nature of the case, and the kind of nurse which is desired, in order to carry out the requisite treatment. It is essential, too, that when the co-operation or agency is unable to supply a particular nurse, the fact should be intimated by telephone or telegram at once. There is no difficulty in regard to the expense where regular clients are concerned, but, in every case the rules should be sufficiently elastic to enable such an intimation to be invariably sent. Unpopularity and loss of business have resulted to some of these agencies from carelessness and want of attention to this matter, and several complaints have reached us from time to time.

In one case a serious accident happened in the country, and the medical attendant telegraphed asking the superintendent of an agency whether she could supply a nurse who had had special experience in cases of severe injury to the head. An affirmative answer was received from the agency. Fortunately the surgeon was a man of considerable experience, so he wired to a friend to go to the agency and see the nurse before she was sent on to the case. It then appeared that the nurse who had been selected had not had any experience whatever in head cases. On inquiry the friend found that there was, however, a suitable nurse available, and further ascertained that she had not been selected because her name did not come next on the list for duty. In this instance the patient was already in charge of two good nurses, but neither of them had had the special experience necessary to give him all the care which the surgeon required if a recovery was to be hoped for. Here the action of the official in charge of the agency might have resulted in loss of life, and this case illustrates how essential it is that whoever undertakes to supply trained nurses to the public shall accept that duty with a full sense of responsibility, and under regulations which are elastic enough to secure that no nurse shall be sent who is not capable of fulfilling the requirements laid down by the practitioner in charge of the case.

We believe that many of the complaints which are made against private nurses have their origin in a failure on the part of the agency to do its business properly. Another serious difficulty which confronts medical practitioners and their patients who require the services of a private nurse in country districts is the failure to reply to telegrams. In a recent case a telegram addressed to a nurses' co-operation received no reply, nor was any nurse sent. Subsequent inquiries showed that the telegram was received, but that, as no nurse was available, the message was passed on to another institution without communicating with the sender. It is perfectly clear that every nursing institution ought to consider the serious dilemma in which a medical practitioner may be placed when the authorities fail to intimate to him whether or not they can send the nurse telegraphed for. If the authorities of a co-operation or agency decline to accept the responsibility of replying by telegram unless it is prepaid, this fact should be clearly intimated in all their advertisements and papers. Possibly such an intimation might not increase the confidence of the medical practitioners and the public, but as a matter of business such an intimation should be given, or a reply should invariably be sent. No business can be successfully conducted unless every attention is paid to the reasonable requirements of its customers.

Failures like those we have been considering have impressed upon the profession the advantage which a practitioner gains by obtaining such private nurses as he may require from one of the hospitals which possess a private staff. These hospitals are thoroughly well organised, and they take every care to meet as far as possible the requirements of members of the medical profession, and also of the public. No well organised hospital would think of sending out a private nurse to a special case, of which particulars were given, unless she had enjoyed the necessary experience to enable her to do justice to it. It is possible that before many years are over the public and the profession will prefer to obtain their private nurses from the hospitals, in which case material changes must take place in the present inadequate system for the supply of private nurses. We have always been in favour of the registration of all the agencies and institutions which supply nurses to the public. If such registration existed many of the abuses and difficulties which attend the present system would be obviated, and the reputation of the private nurse would be liable to suffer less than at present from causes for which the nurse herself is in many cases not directly responsible.

### Abdominal Surgery.

By Harold Burrows, M.B., F.R.C.S., Assistant Surgeon to the Seamen's Hospital, Greenwich, and to the Bolingbroke Hospital, Wandsworth Common.

### AFFECTIONS OF THE STOMACH.

### Complications of Gastric Ulcer.

*(Continued from p. 33.)*

*Perforation.*—When an ulcer has extended through the walls of the stomach so that an opening is formed between the inside of the stomach and the peritoneal cavity, the ulcer is said to have perforated. The complication is caused more frequently by acute ulcers than by those which are chronic. The results are serious, and unless the patient is operated on within a few hours of the onset of symptoms he is almost sure to die of peritonitis.

The symptoms are those of peritonitis, and are usually characterised by the suddenness with which they appear and the rapidity with which they progress. The patient is seized with intense pain in the abdomen, and suffers from retching and vomiting. His face soon displays an "abdominal expression," the pulse becomes rapid, and unmistakable signs of grave abdominal trouble appear.

Fig. 6.

The shaded area (umbilical region) is the most frequent situation of abdominal pain.

Fig. 7.

The shaded portion represents the area of tenderness in the early stages of perforated gastric ulcer.

The chief difficulty in diagnosis lies in distinguishing between perforated gastric ulcer and peritonitis secondary to some other lesion, especially acute appendicitis. An accurate history of the case will often be a help in making the distinction, for if the patient has had previous attacks of pain in the abdomen with intervals of complete health, the probabilities in favour of the pain being due to appendicitis are increased. On the other hand, the absence of such attacks, and a history of indigestion, point to a gastric ulcer as the origin of his troubles. Sometimes, and especially in a hospital, the nurse has opportunities which are denied to the surgeon for questioning the patient's relatives on these points; and with a view to obtaining all the evidence possible it is advisable that the nurse herself should make some inquiries as to the patients' history. This remark applies to all acute abdominal cases; and, especially with female patients, the nurse may be of the greatest assistance to the surgeon in eliciting information from the patient or her friends.

Apropos of the stress I have laid on the difficulty of diagnosis in these cases, it may be wondered how pain originating in the stomach which is in the upper part of the abdomen, can be confused with pain originating in the appendix or the pelvic organs, which lie in the lower part. The answer is that pain is a treacherous guide. A patient with a perforated gastric ulcer may feel all his pain in the right iliac region—that is to say, in the neighbourhood of the appendix—and a patient with appendicitis may refer his pain to the umbilical or epigastric regions.

Tenderness to pressure is reliable in the earliest stages, but soon the entire abdomen may become tender so that no "localising" symptom remains.

As stated above, the only treatment for perforated gastric ulcer is operative, and the sooner this is carried out the greater will be the likelihood of success. If twelve hours have been allowed to slip by between the accident and the operation the patient's chances will be rapidly dwindling away, and at the end of twenty-four hours operation will probably be too late.

It follows that the preliminary treatment of a case of perforated gastric ulcer will include preparations for an immediate operation. As to the patient himself, he should be kept quite still on his back. Hot flannels applied to the abdomen may ease his pain a little. On no account should anything be given by the mouth—not even water. Morphia, opium, and other sedatives are most mischievous at this stage. Without helping to cure the patient, they alleviate his pain and so disguise other leading symptoms that the gravity of the illness is apt to be overlooked, and operation delayed until it is too late. When once the condition has been diagnosed and operation decided upon, these objections, of course, no longer hold good. The most important aim previous to operation is to keep the patient at perfect rest in one position. He is not likely to move about of his own accord, but in taking him from his home to the hospital, from one room to another, or from his bed to the operation table, a great deal of harm may be done. It is not difficult to appreciate the fact that when a gastric ulcer perforates there is an escape of some of the stomach contents into the peritoneum, causing at first a local peritonitis only.

But if the patient is rolled about and doubled up, as frequently happens during the manipulations mentioned above, the stomach contents which have escaped into the peritoneum are given the opportunity of being distributed throughout the abdomen and causing general peritonitis.

This matter has received little or no attention in the text-books, and therefore I am anxious to lay great stress upon it. Not only in cases of perforated gastric ulcer, but in all acute abdominal disorders, it is essential to keep the patient on his back as still and as rigid as possible from the onset of acute symptoms until the operation is performed. The common practice of sending a patient who is suffering with an acute abdominal lesion to hospital propped up in a cab cannot be too forcibly condemned.

The operation for perforated gastric ulcer consists essentially of opening the abdomen, sewing up the aperture in the stomach wall, inserting drainage tubes to remove stomach contents and products of inflammation from the peritoneum, and sewing up the abdominal wound. There is nothing special to note in the after-treatment.

*Gastric Adhesions.*—The inflammation brought about by an ulcer may cause the stomach to become adherent to neighbouring organs; so, too, may inflammatory conditions of these latter cause them to adhere to the stomach. In practice the chief causes of gastric adhesions are ulcer of the stomach and cholecystitis (inflammation of the gall-bladder).

The chief symptom is pain after food, and the condition is difficult to distinguish from simple ulceration. In some instances the adhesions may prevent the stomach from expelling its contents into the duodenum, thus giving rise to symptoms of pyloric obstruction. There are two operations which may be carried out for the remedy of symptoms due to gastric adhesions. The first is gastrolysis, which means division of the adhesions; and the second is gastro-enterostomy, or the making of an artificial opening between the stomach and intestines, so that the stomach is provided with an alternative exit for its contents.

### The Nurses' Clinic.

### DYSENTERY AND THE REST CURE.

Anæmia is a very frequent complication of dysentery. If it is severe the patient may be ordered a modified rest cure, lasting from three to six weeks, according to the progress he makes.

He will rest in bed altogether, though he will probably be allowed to have a bath once a day and to see a few friends. Nourishment must be taken every two hours, as much change as possible being made so as to tempt his appetite. The diet will consist at first of milk—peptonised or diluted, according to the doctor's orders—Benger's food, arrowroot, rusks and milk, raw meat sandwiches, and raw meat juice; but as he improves and his powers of digestion become stronger his food can be increased until he is able to eat ordinary meals. Intra-muscular injections of arsenate of iron are sometimes given in these cases. The syringe used is made entirely of glass, and about 15 minims of the arsenate is injected into the muscles of the buttock every other day. The nurse should have ready, in sterilised water, the syringe and needle (the latter should be wrapped in lint before being put in the steriliser, so as to protect the point), a bowl of lotion and some swabs for cleansing patient's skin, some ether or ether-methylated for the same purpose, and a collodion dressing for application directly the needle is withdrawn.

The room in which the patient does his "rest cure" should, of course, be quiet, light, and airy—the more fresh air he has the better. His clothing must be warm, and if he is allowed to go to a bathroom for his daily "tub," care must be taken that he does not dawdle in passages on the way, and also to see that windows are shut and the bathroom is warm.

One treatment for dysentery—that of Drs. Dapper and Von Noorden—consists of giving the patient a diet of meat, stringy vegetables, crisp toast, biscuits, fruit, cream, and butter, tea is allowed, but it must be freshly made and not allowed to stand, and nothing should be drunk until the meal is nearly over. The meals should be regular and punctual, and no food need be taken between them.

It requires some courage on the patient's part to begin this treatment, especially if he is just recovering from an acute attack, and the nurse must do her best to cheer him up and to make him really believe that not only will it do him no harm, but that he will soon be rewarded by complete recovery.

Relapses may occur, but he must be urged to persevere and not to get despondent about himself.

Another course of treatment consists of giving the patient large doses of ipecacuanha while keeping him on low diet.

The patient is kept in bed entirely and has feeds of milk and barley water every two hours, from 6 A.M. until 6 P.M. At 9 P.M. the ipecacuanha is given, after which he must lie flat on his back without pillows, and without speaking or moving, until all fear of vomiting is past. He should be provided with plenty of swabs for wiping away any saliva.

Twenty minutes before the first dose of ipecacuanha is given the patient is generally ordered some tinct. opii, and the application of a mustard leaf to the epigastric region to assist him in keeping the medicine down, and this is repeated on the following nights if there is any sickness.

The amount of ipecacuanha given is gradually reduced and the diet increased, until in about a fortnight only 10 grains are being taken—5 grains at bedtime and 5 grains in the early morning, fasting.

At the end of ten days he may be allowed to sit up in bed a little, and soon after to get up, but care must be taken that he does not become tired and that he is warmly clad.

In some cases the vomiting caused by the ipecacuanha is so severe that the "cure" has to be abandoned, though in others there is little or none.

Diarrhœa also may be caused, but this is not likely to be serious.

The patient's room must, of course, be kept very quiet, and no visitors should be allowed until he is quite convalescent.

In chronic cases rectal injections of solution of nitrate of silver are sometimes ordered. They are generally given three times a week, after the bowels have acted. The amount given is from two to four pints, and it should be administered slowly through a tube and funnel.

The strength of the solution will, of course, be ordered by the doctor, and great care must be taken in using it, as should any be spilled either on the bedclothes or furniture the stains cannot be removed.

The result of the injection must be attentively examined for blood, mucous, casts, etc., and, if necessary, kept for the inspection of the doctor.

### Incident in the Life of a District Nurse.

COMPANION WATCHERS.

One very cold day in the middle of winter, while the snow in Scotland lay several feet deep on the ground, word was sent up by the parish minister that he wished me to go and see an old man who was very ill. This old man, whom I knew by sight, lived about four miles away from my lodgings. After having dressed and provided myself with a wrap, I set out to trudge through the deep snow accompanied by a strong walking-stick. Having to go down a steep hill from the house where I lived, I several times almost disappeared in deep drifts, the snow sometimes being up to my waist. But at last I found that I had reached the road, and I heaved a sigh of relief as I proceeded on my journey.

At last I reached my destination and was shown into a dark room filled up with all sorts of rubbish: old boxes, several broken chairs, an old chest, old rat traps, and various articles of no use whatever, whilst dirt, poverty, and misery were all around. In a very dark corner I discovered a bed —or what was meant for such—a few boards formed into a long box. Over this was some dilapidated straw, and on this lay the old man, whom I discovered in his outdoor clothing lying in rags and filth. Getting off my cloak, etc., I proceeded, with the aid of a neighbour, who had been in attendance till my arrival, to get the old man's clothing off, and to make him a little more comfortable.

Time wore on, and no arrangements having been made for my comfort, which is often the case in this part of the world, I began to feel a little hungry, and at last I took matters in my own hands, procured the necessaries from the nearest shop, prepared my tea, and sat on a chair with a newspaper for a cloth, and an old cracked cup without a saucer for drinking out of. However, under the circumstances I managed to enjoy my humble fare. A little later there were several anxious inquirers as to the patient's state, but as evening wore on they gradually diminished and at last everyone cleared off, including the old neighbour who had acted as helpmate. She informed me "that she didna sleep for two nights and two days watching the old man, but that since I had come she would go away as there was no need for her." Truly, I would have been glad of her company. However, it being 10 p.m. then, I reconciled myself to a long winter night's watch. To make things worse, the only window had two or three of the panes broken out, and the wind and snow came right into the middle of the room, which, of course, did not tend to make the place warmer. The only light I had was from a small halfpenny candle and a very poor fire, which feebly illuminated the miserable looking sick-room. About midnight I had plenty of company, but not of a desirable nature. Rats as big as rabbits ran about in dozens. One bold horrid-looking veteran actually came over to the fire and sat looking me in the face, till at last, not being able to stand it any longer, I lifted a piece of coal and flung it at him as hard as I could. The wretch scampered off behind the old chest, giving his head a fearful bump in the exit, and as I do not remember seeing him again I hope that the damage was serious. Two nights and two days passed in this way. On the third night the old man got worse. At about eleven o'clock there was rustling of the straw on the bed. Thinking to myself that the patient was getting restless I came over and watched him carefully; to my horror saw several pairs of eyes looking at me in a bold fashion through the straw! The old man, though evidently dying, noted my terrified face, and said in a whisper, "Nurse, take that big stick and keep it in your hand to scare them away; I always have it beside me—I caught thirteen the other night in the traps." As I did so, I could not help thinking what a life he must have lived, poor old man! On the fourth night, about 8 p.m., he died. With the aid of the old woman, I washed and dressed the body, but though she visited me now and again during the long hours, most of the night I had to sit alone with the stick in my hand to keep those awful rats off the corpse. Next morning the few relatives having arrived, I set off home to walk the four weary miles between here and my lodgings, which I reached in a sad state of exhaustion and fatigue after my trying, and, I hope, unusual experience. Never was I more thankful to get back to my own rooms, which at least were cheerful and clean.

### "The New Probationer."

The nurses' tea-bell had clanged through the busy city hospital. Already nurses were issuing from their wards in the different corridors, waiting one for another with the day's gossip, and looking for the most part tired and jaded, for it had been a dull wet day, and "receiving day" into the bargain.

Presently out of one of the medical wards stepped a new probationer, hesitating somewhat, as though not quite sure which way to turn. Many curious eyes watched her as she paused, for she was very beautiful—perhaps never had a more beautiful girl worn uniform. She was tall, slim, and graceful, with a pale, proud face, that would have looked more in keeping with a coronet than the stiff linen cap she wore, and more than one nurse gave an envious sigh as they looked after her.

"Who is she?" asked a Resident of two nurses he had overtaken.

"She is the new nurse in your ward," answered one of them.

"What a pity I change into the surgical side to-morrow," said the resident, with a laugh and a shrug; "and dear old short-sighted Eames, who takes my place, will never notice if she is pretty or not," he added regretfully.

He spoke carelessly, and with a nod separated from the nurses at the dining-hall door.

During tea the two nurses watched the new probationer. She was gazing round her with an abstracted air scarcely flattering to her surroundings. But how were those watching her to suspect that she had assumed a mask to hide her deeper feelings? How were they to guess that under that brave front lay a heart throbbing with a dull deep sense of disappointment, and a sickening realisation of unfittedness for the work undertaken. She had heard so much of nursing—of the busy, happy life. She had waited such an age to be admitted into this particular hospital, and now she was conscious of only one desire—a craving to go home again, to be one of that happy family circle that had let her go so unwillingly. She thought of the ward, with its dull city outlook and its depressing atmosphere. She thought of the sickening sights she must face, and of a sudden her nerve failed her. Could she, she asked herself accusingly, endure such a life for three long weary years? Appalled by this awful thought, she went back slowly to her ward. As she entered it the Resident who had addressed the two nurses was showing his successor his cases. She was struck by his companion's unusual height and by the thickness of the glass in his spectacles; then she forgot them in the midst of her own misery. Still in a reverie, she began her evening's work. Taking a bowl of water to a bad-heart case, she asked if she might wash him.

The man smiled, unused to such politeness, and with difficulty tried to undo the button at his throat.

His helplessness roused the girl to a sense of her duty.

"Let me do that," she cried quickly, and then, as she washed his lined, suffering face, she added, "Do you never grow tired of your life?"

He looked up with a sigh. "It ain't no use, Miss," he gasped, "I've got to bear it, but do you think you'll like being here, Miss?"

The "Miss" was a tribute to her youth and beauty. It seemed to this rough man a liberty to call this beautiful stranger "Nurse." "I hope I may in time," she answered hopelessly. "As yet everything seems appalling; I mean it is all so new and strange, but no doubt I shall get accustomed to it in time." The clumsy Resident taking notes of his new cases at the next bed overheard her words and felt a sudden thrill of compassion for her, and looking up at that moment, she encountered his eyes and read in them his pity. His look haunted her, and as she went to refill her basin, she said to herself, "I am glad he is going to be our Resident; he looks sincere and a gentleman in spite of his ugliness." The next patient to be washed was a young boy—weak and exhausted by pneumonia. He worked in a colliery, he told her, and was amazed to hear that she had been down a pit and knew the dangers of the life.

"Is your father a miner?" he asked with a glow of interest. The probationer did not smile. "No," she answered, "but he often goes down a coalpit, and would be very interested to meet you."

"I expect he has a pal a miner," said the boy in a questioning tone.

"He has many," said the probationer with a lovely smile.

The clumsy Resident, prolonging his case, lost none of her words. The good breeding of her answer thrilled him, for his was a sensitive nature that could not wound. Once the girl laughed at something the boy told her, and it was a mirth-provoking laugh, with a sadness underlying it that hurt him. At that moment the staff nurse corrected her sharply for her slowness, and the Resident felt a savage desire to hurl his case-book at the older woman's head.

The next morning when he woke he was conscious of a feeling that he had not known for years. A sense of expectancy took possession of him. The new probationer was being scolded when he entered the ward, and he wished himself miles away.

"I don't know why the matron sends me all the fine ladies to train," the staff nurse was exclaiming. "I'm sure this is the heaviest ward in the house. At least she might give me good workers."

The girl's proud face flushed scarlet at her words, but no sound escaped her till the staff nurse finished her lecture, then "I am sorry," she said, "I did my best."

"I suppose you did," snapped Nurse Baker. "But let me tell you you'll have to do better if you intend to remain in this ward."

The clumsy Resident decided to interrupt the conversation. "Good morning," he said with an awkward bow that included both. "Isn't it wet to-day?"

Nurse Baker was too angry to reply, and the probationer by an unlucky impulse answered for her. "It is indeed," she said, turning. "A most depressing day."

"You'd better give No. 10 his medicine," snapped Nurse Baker, "and then go off duty."

It seemed to the Resident that the probationer's eyes spoke of unshed tears, and he thought of her all the time he was going his round.

He pitied her intensely in the days that followed, when he saw how bravely she tried to get accustomed to the hard life and Nurse Baker's faultfinding. He grew to detest the staff nurse when he saw how she delighted in humiliating the proud, spirited girl in the presence of doctors and nurses, and strove to show his pity by offering her little polite attentions.

"You must not mind Nurse Baker," he told her once when the staff nurse had been unusually unreasonable. "It's only her way, she really means it for your good."

"It's not Nurse Baker," the girl answered wearily. "It's—it's the patients. I'm quite ashamed to own it, but I shrink from them more and more. I wish I had never come here. Oh, how I wish I had never come."

"But you never show your feelings," he said gently. "All the patients adore you, so what does it matter when they never detect your feeling?"

"But I am miserable," she cried. "I never had anything to do with the poor before, and I'd run away only I hate to be beaten."

"You won't be beaten," he told her gently. "Believe me you will grow accustomed to the sights and the poor in time, and learn to pity them; poor things, they appreciate sensitive delicate-minded nurses just as much as their superiors do."

That night he decided to try and help her. I will get her removed to the children's ward, he said to himself, and went to the matron. The matron, who liked him, consented to the change, and he carried a dull heart, but (what he thought would be) glad tidings to the new probationer.

She was singing when he entered the ward a verse from "Bonnie Mary of Argyll," and the sadness of her voice made him hurry to her with his news, and looking down at her from his great height he told her of the proposed change.

"It will be of no use," she said despairingly, "I am not one bit suited to be a nurse. I ought never to have come, I am always selfishly longing for the things that used to make up my life—for dances and theatres and hunting. You don't know how I miss my horse." She ended with a weary sigh that hurt him more than any complaints could have done. Suddenly she looked up entreatingly. "Do not blame me," she cried impulsively, "I tried to be kind and unselfish like you. I tried to like the men; then last night I grew so homesick, and I wrote to mother and asked her to send father to fetch me home."

Even as she spoke, the ward door opened, and the matron ushered in an elderly gentleman. He was a tall, distinguished looking man, with iron-grey hair and kind grey eyes.

"Well, little woman," he said, taking his daughter's hand in both his own and warmly kissing her. "So you have decided to come home. I need not tell you that we are all glad." Then with a charming courtesy he turned to the Resident: "My daughter has told us of your kindness to her," he said. "May I thank you? The matron," he continued, "says Constance may leave to-morrow morning, which I think very kind of her."

Dr. Eames mumbled a suitable reply and excused himself. Like one in a dream he passed down the stairs and out into the lamplit streets, quite oblivious to the heavy rain, and to the fact that he was bareheaded and in slippers.

On he went, peering through his spectacles, thinking of the girl and her father. They were not of his world, he reminded himself. They belonged to that exclusive circle he would never enter. "Idiot that I am," he thought bitterly, "to have built such a fool's castle." Suddenly he was conscious of hoarse cries, of women waving and shouting to him, of a horse's nose thrust on his shoulder. He looked up, dazed and bewildered, tried to save himself; but in vain, a fire engine loomed above him, he felt himself thrown down, something passed over his shoulder, and he knew no more. Someone in the crowd recognised him. They carried him back to the side room of his own ward, and there the new probationer saw him lying, covered with blood and unconscious. Not by the flicker of an eyelash did she betray her felings, but something gripped her heart and drove the blood from her face; it was not horror, not pity; the feeling was a new one, and she paused to reflect.

"Will he die?" she asked the surgeon who was examining him. He looked up amazed at her presumption. "No," he said shortly. "He will live, but he will want careful nursing." Then of a sudden it came to her that she could repay all he had done for her. She rose to her feet with a look of resolve in her eyes, and made her way to the matron's room.

"I have changed my mind, Miss Crump," she said. "I want to remain with you and in the same ward if I may. It was cowardly of me to want to go home. Will you excuse me and let me remain?"

"Certainly," said the matron kindly. "I thought it was only a momentary sense of home-sickness; you will make a good nurse. I told your father so, and no one will train you better than Nurse Baker. By-the-bye, how is poor Dr. Eames?" It was well the room was badly lighted, else the matron must have noticed the flush that mounted to the girl's face as she answered:

"Mr. Fawsitt says he will live."

"Thank God," said the matron as she dismissed her with another kindly smile.

An hour later the new probationer stood alone in the side room where Dr. Eames lay. The light was low and the unconscious man breathed heavily. The girl hesitated, glanced round her sharply, and then like a thief stole to the bedside. "I shall not be allowed to nurse you," she said in a whisper. "But I don't mind now, for some day I will tell you that I stayed here to please you, and you will be glad."

### Nursing in a Monastery.

I HAD just finished my training in one of our large hospitals, and had joined a well-known Nurses' Home in the North of England with a view to gaining some experience in private nursing. The sister-in-charge called me into her room on the afternoon following my arrival at the home, and told me to be ready in two hours' time to go by train to a monastery, my patient being one of the monks.

After a rather tedious and tiring journey I arrived at a small country station four miles from my destination. The drive was a most beautiful one, through an exquisite tract of country. The monastery was quite a huge pile, and looked very picturesque illuminated by the rays of the setting June sun.

I was shown into a fine oak-lined hall, and from there was guided to my room by a porter or door-keeper.

Just as I had finished removing the dust of my travels, and had changed my dress, a knock came to the door. On opening it I was confronted by a short jovial-looking man in a Benedictine habit. He informed me that he was the "Infirmarian," and proceeded to guide me to the Guest House, where my patient was lodged. Members of my sex not being allowed in the monastery, he had been removed from his room there.

My patient proved to be a young man of about 27, gaunt and emaciated to the last degree, suffering from an attack of appendicitis. I learnt afterwards that he had recently finished the probationary and extremely severe two years' training for the priesthood.

The room he occupied had evidently at some far distant period been a small chapel or oratory, having a most lovely triple stained-glass window at one end.

Through the open lattice came the strains of an organ, and the sound of men's voices chanting. The Monks were singing the office of Vespers.

All the night long I busied myself with my patient, who was very ill, and suffering intensely. Towards 4 A.M. just as the sky towards the East was assuming a rosy tint, a bell began to ring. Opening the door into the corridor, which was an extension of the cloister, I saw in the dim morning light numerous dark figures with cowls drawn over their heads passing noiselessly on. The religious were on their way to the church to say Prime, the first office of the day.

During the course of the morning I was welcomed by the Abbot and the Prior, the former a saintly looking old man, with a face in which sweetness and strength were wonderfully combined. He wore over his habit a long gold chain and a crucifix.

Previous to taking some rest I went out for a walk in the precincts, and was charmed by the exquisite and extensive view stretching from the terraced front of the monastery. A more ideal spot could not be imagined, and to one coming as I did from the constant toil and bustle of a large hospital, the peace and beauty were idyllic.

While on my tour of inspection I visited the church, which was empty at that hour. I found it to be quite a modern building, with a most beautifully-sculptured stone sanctuary screen. Round the building, and separating the different side chapels from the nave, the stations of the Cross were carved in the same beautiful stone.

The life of these Benedictines is a full and busy, as well as a healthful, one. Their recreations are principally botany, natural history, and geology. The lay brethren do the housework and cleaning in the monastery, and much of the manual work outside.

The Abbot's hobby is poultry farming, which he carries out very successfully.

The feast of Corpus Christi was kept during my stay, the procession round the grounds, headed by the Abbot, under a gorgeous canopy, being most impressive.

During my sojourn among those Benedictines I could not fail to be struck by the kindness, consideration, and deference which was shown to me, a Protestant,

At the end of a month my patient had entirely recovered, and I left the quiet and beautiful retreat with much regret, gladdened by all the kindness which had been shown me, and refreshed by the quiet beauty and the pure air of my surroundings.

### To Nurses.

WE invite contributions from any of our readers, and shall be glad to pay for "Notes on News from the Nursing World," "Incidents in a Nurse's Life," or for articles describing nursing experiences at home or abroad dealing with any nursing question from an original point of view, according to length. The minimum payment is 5s. Contributions on topical subjects are specially welcome. Notices of appointments, letters, entertainments, presentations, and deaths are not paid for, but we are always glad to receive them. All rejected manuscripts are returned in due course, and all payments for manuscripts used are made as early as possible after the beginning of each quarter.

### A Surprise Visit.

### COUNTY COUNCIL INSPECTION OF A MIDWIFE.

"Good afternoon, nurse! You are the district nurse and midwife, are you not? "Very lucky for me to catch you at home. I had not time to let you know I was coming. I am the medical officer of health of the County Council, and have come to inspect your bag, appliances, books, and certificate. May I come in? I will not keep you long."

So spoke the courteous medical officer of health to me one afternoon, a short time back, just as I was starting off on my afternoon round, and I realised that the dreaded inspection of my basket, appliances, etc., was to be made, without any preliminary notice having been given!

Having invited my superior officer in, he asked me first to let him inspect my basket and appliances. Everything was taken out and thoroughly examined to see if in working order, the bottles looked into, and a question or two asked as to when ergot was given.

The basket lining of holland was also well inspected, and its method of changing the lining shown. My catheters (rubber and glass) are kept in a fold of lint in a small tin sandwich-box. This was thought a good idea.

My steriliser (a fish kettle) was next seen, and questions asked about sterilising.

Next, my book of cases was examined to see that it was kept in accordance with rules. A few questions on the Central [illegible]idwives Board rules were asked me, chiefly to see if I un[illegible]ood them; and especially when to decline to attend [illegible] alone.

Nex[illegible]entral Midwives Board certificate was looked at, its [illegible]r and examiner being jotted down.

My [illegible]icer told me to write him at any time respecting rules [illegible] any difficulty, and he would be pleased to help me and [illegible] me right.

He also offered to explain anything to me that I wished, and after a little more talk about my general and midwifery training, with comments upon his own midwifery training, he affixed his signature to my last case, and rose to go. Thus ended my first inspection by the County Council, an ordeal which no nurse could have any cause to dread. In fact, if all inspections are conducted as pleasantly as mine was, they should prove a distinct help to a district midwife.

### Everybody's Opinion.

[Correspondence on all subjects is invited, but we cannot in any way be responsible for the opinions expressed by our correspondents. No communication can be entertained if the name and address of the correspondent are not given as a guarantee of good faith, but not necessarily for publication. All correspondents should write on one side of the paper only.]

### SEAWEED FOR A SPRAIN.

"Topsy" writes: "While rambling over some rocks at the seaside, I slipped and sprained my wrist. I immediately bound it with broad seaweed as a support, and used it as a dressing for a few days. It gave great relief, and after a short time it got quite well. I think that it might prove useful to other visitors at the seaside."

### WORKING UNDER HIGH PRESSURE.

"An Infirmary Sister" writes: I should be interested to know if others have to work under such high pressure as I do. I have an imbecile and chronic ward of about thirty patients and over. I have only one "probationer nurse." It is impossible to have work done as it should be done, with so many old and helpless sick ones, with so little help. I am given to understand that the Local Government Board grants one nurse to every twenty patients, but does the Board mean helpless sick ones?

### OPERATIONS ON MALE PATIENTS.

"A Member of the Guild of St. Barnabas" writes: In reply to "A. B. C.," who writes from Newfoundland, one might advise that she would use her own common sense. To the pure all things are pure, and to a nurse all parts of the body are sacred, no matter where an operation has to be performed. "A. B. C.," I trust, will gain experience as she gains in years. May she never know with what indignation this question has been read by many trained nurses.

### MATERNITY NURSES AT A DISCOUNT.

"A. C. M. B." writes: Having read your note on "Maternity Nurses at a Discount," in your issue of April 21, I beg to offer a few words in their favour. I consider that a nurse who holds a certificate of three years' training, and in addition a certificate for monthly nursing, is fully qualified to undertake a monthly case, and no medical man has any right to expect her to possess the certificate of the Central Midwives Board. No doubt it is preferred, especially by people who aim at getting the most for their money. Are we to suppose that in the future it may be insisted upon? If a lady cares to engage a midwife in addition to her medical man she must be prepared to pay accordingly. A midwife is justified in asking for a midwife's fee. The expense incurred in the training for the examination of the Central Midwives Board is no small matter, and the fee of an ordinary monthly nurse is as a rule exceedingly small. It is unfair for a fully certificated monthly nurse to be refused work simply because she does not possess the qualification of a midwife.

### A PLEA FOR HOBBIES.

"E. L." writes: Every nurse ought to have a hobby. That is, it is essential that she should have some absorbing interest outside her work, something sufficiently interesting to make her forget it for the time. At the present day we have excellent nurses, but a great number of these, really well-trained and capable women as they are, suffer from one-sidedness. Part of their nature has been over-developed to the neglect of the rest. They have given too much of themselves to their work. Nursing is absorbing, and it does make tremendous demands on its votaries. It absorbs time, thought, energy, ability, affection, to the exclusion of all else. This makes for narrow-mindedness, which is one of the great failings of nurses as a class. It might tend to restore the balance a little if each nurse were to keep on any hobby or pursuit in which she had taken an interest in her pre-hospital days. She would not find it an undue tax on her strength. Absence of occupation is not rest, change of occupation sometimes is, and a hobby that compels a fair amount of attention and thought and even energy, would prove the greatest boon a nurse could have. By taking her thoughts completely away from her work and her patients, she would be enabled to return to her ward and its duties braced up and strengthened beyond belief. What is even more important, her character, the real ego, would gain immeasurably.

### WAITRESSES ATTIRED AS NURSES.

"Progress" writes: There are so many complaints from all quarters of the nursing world concerning the abuse of the nurse's uniform that I venture diffidently to suggest a solution of the problem. I would like to point out that it is simply the fact that the nurse's uniform is so becoming to the wearer which tempts persons not belonging to the profession to adopt it. Whoever heard of the uniform of a Sister of Mercy or a Salvation Army lass being copied? I used to think both extremely ugly. Now I see the wisdom of their adoption. These uniforms are not becoming, but they are distinctive. I propose in all seriousness that some of the heads of the profession should design a uniform which could not be easily copied, and one which would not fill the average nursemaid or waitress with envy; that its manufacture should be entrusted to the particular firms which cater for nurses, and who would undertake to supply the same only to them. Thus we could retire gracefully from our present equivocal position, and gain, I think, a great deal.

---

"Nurse F." writes: I daily watch for wearers of our uniforms, and read with interest the letters on same. I think it is a great pity that people who employ nurse-girls and nurses for their children have not more respect for our uniform, as it is connected with pain and sickness, and should be held sacred. It is mockery for girls to wear dirty untidy white strings, and bonnets shabby, as nearly alike as they can procure, and certainly the owners of the restaurant to which reference has been made could prevent the indignity they encourage. District nurses are obliged to wear uniform. I have a medal with "R. M." in silver and a scroll at the bottom, and I have not found anyone copy me yet. The medal can be had with the letters "T. N." on, and the name of the hospital at the bottom at which the nurse was trained. The letters mean registered midwife and trained nurse.

---

TRAINED NURSES AND UNTRAINED WOMEN.

"F." writes: I should be glad if I may be allowed to say how much I agree with what a "St. Thomas's Nurse" said a few weeks ago about untrained and half-trained women wearing the same uniform as fully trained nurses. I belong to one of the large London hospitals, and we have had a good laugh over the grievances of the only too apparently half-trained women who have been airing their opinions these last two weeks in our paper. I say "only too apparently half-trained women," because were they fully trained and doing right in wearing the uniform, they would be as jealous of it as I am, anxious that no one should behave in such a way as to disgrace it. They have, too, quite misunderstood "St. Thomas's Nurse," who did not say that no nursemaid makes a nurse, but that no nursemaid ought to be allowed to wear the uniform of a nurse unless trained. I think that it is bad form to wear uniform, if you can possibly do without it, and so say all West End doctors and nurses. Something might be done, in the way of having a medal struck, only obtainable upon producing the certificate: the colour might be different for the three years and over, the two years, one year, and fever and maternity nurses; then the public would know who were sick nurses and who nursemaids, etc. Some people say "I should not trouble to complain of all these people wearing your uniform, even if they are not trained"; but I only wish that every nurse would so rouse herself that there might be a way found of protecting our cloak and bonnet; instead of being content to urge, as a policeman did last week to an American, when asked why the dying horse was allowed to linger in agony; "It's not my business."

---

NURSES AND TESTIMONIALS FROM MEDICAL MEN.

"A District Nurse" writes: I should like to suggest on behalf of some of my nursing sisters as well as myself—whose hospital training on account of family or other matters has necessarily been short—that, although so much value is now attached to a three years' certificate, surely testimonials from medical men and others who have had opportunity of judging of the capabilities of a nurse, ought to be taken into consideration. There are qualifications—other than those obtainable in hospitals—which are absolutely necessary for a district nurse, without which it is impossible to work with success amongst the poorer classes—*e.g.* kindly sympathy, tact, self-sacrifice, and patience. It is one thing to be in the hospital with medical skill and help and everything necessary at hand, and very different to be in a country district, perhaps miles from a doctor, and having to make the best of what the cottage contains, which, alas! is oftentimes very little. I think that as a rule the nurses who have been so long in hospital do not manage as well as those who have had shorter hospital experience, no doubt because they are so accustomed to having everything at hand that is likely to be wanted. There are undoubtedly many nurses at the present time, whose heart is in their work, and who have had years of district experience and hold good testimonials from doctors and others, but who find it difficult to obtain a post because they do not hold a three years' certificate. It seems hard that successful workers should have to withdraw from the ranks on that account. Nursing, like all other professions, needs the whole heart and soul put into the work to make it a success, and I cannot resist the conviction that if those who wish to make it a life work would first count the cost and then enter from the highest motive we should have helpful and encouraging correspondence, which, instead of making outsiders look upon nurses as "a grumbling, discontented lot" would raise the standard in the eyes of the world, as a noble and real desire to benefit fellow creatures, and would be the means of helping, instead of hindering, the nursing profession.

---

### Appointments.

[No charge is made for announcements under this head, and we are always glad to receive and publish appointments. The information, to insure accuracy, should be sent from the nurses themselves, and we cannot undertake to correct official announcements which may happen to be inaccurate. It is essential that in all cases the school [illegible]ining should be given.]

---

Borough Hospitals, West Bromwich.—[illegible] Faith Wilson has been appointed junior charge n[illegible]he was trained at the Royal Infirmary, Bradford, and [illegible]e been nurse at the Bradford Nurses' Institution and the [illegible]yston Hospital.

Grimsby and District Hospital.—Miss E. Dav[illegible]s has been appointed matron. She was trained at the Royal Hospital, Portsmouth, and has since been staff nurse at the Children's Hospital, Newcastle-on-Tyne; staff nurse at the North Devon Infirmary, Barnstaple; staff nurse at Poplar Hospital for Accidents; sister at Cumberland Infirmary, Carlisle; sister at Huntingdon County Hospital; sister, and subsequently night superintendent and home sister, at Bristol Royal Infirmary; and matron of the Royal Cornwall Infirmary, Truro.

Reigate and Redhill Hospital.—Miss Irene Thomas has been appointed staff nurse. She was trained at the Park Hospital, Hither Green; the Hospital for Women and Children, Waterloo Road, London; and the Royal Hospital, Portsmouth.

Rotherham Hospital and Dispensary.—Miss Holbrooke has been appointed sister. She was trained at the Middlesex Hospital, London, and has since been attached to the Nurses' Co-operation. She has also been sister at Bootle Borough General Hospital, at the City Hospital, Birmingham, and at Lodge Moor Hospital, Sheffield.

Rotherham Union Infirmary.—Miss M. Seville has been appointed superintendent nurse. She was trained at the Chorlton Hospitals, West Didsbury, Manchester. She has since been sister at Sculcoates Infirmary, Hull, and done private nursing on the staff of the West Riding Nurses' Co-operation, Rotherham.

South Shields.—Miss Annie Binks has been appointed inspector of midwives and health visitor. She was trained at the Hospital for Sick Children, Newcastle-on-Tyne, and at the Glasgow Maternity Hospital. She holds the certificate of the Central Midwives Board.

### Presentations.

HIGH WOOD SCHOOL, BRENTWOOD.—On Thursday last week Miss Emily Long, assistant matron at the High Wood School, Brentwood, under the Metropolitan Asylums Board, on leaving to be married was presented with a silver tea service by the matron and staff. Miss Long carries with her all the good wishes of the staff for her future.

### TRAVEL NOTES AND QUERIES.

BY OUR TRAVEL CORRESPONDENT.

A FORTNIGHT IN HOLLAND (Pug).—The trip cannot be done in Holland on the terms you mention. The only way I can suggest is that you should go to Bruges in Belgium, and remain there for the fortnight, making small excursions round about. The cheapest way of reaching Bruges is by the General Steam Navigation Company's boats from Tower Bridge to Ostend, first return 10s. 6d., second return 9s. Rail from Ostend to Bruges, about 1s. 2d. each way. Write on a prepaid return letter card to The Reverend Mother Superior, Couvent de la Retraite du Sacré Cœur, Cour des Princes, Bruges, and ask her if she can take you at the date you mention, and what her terms are. If you work for your living in any way mention it, for it makes a difference.

MUNDESLEY OR SHERINGHAM (Carnation).—We do not undertake to give addresses of lodgings. Write to the Traffic Manager of the Great Northern Railway, King's Cross Station, and ask him to send you the company's Holiday Gazette with lists of farmhouses and lodgings. Enclose stamp.

A FORTNIGHT IN SWITZERLAND (Omar).—Your question is such a wide one that I cannot answer it without more precise information. Write to me again at once, and tell me exactly what you can each afford for your holiday, and I will plan you a little tour to come within the stated sum.

THREE WEEKS ABROAD (Cigogne).—I fear that your terms are too low for most places, though being a party of five or six you may be able to make a satisfactory arrangement. You can travel to Belgium, as you say; it is 10s. 6d. first return and 9s. second by that route. I should suggest three alternative tours. First: ten days at Knokke, seaside, from which you can make some interesting excursions into South Holland, and ten days at Bruges. Second tour: Brussels for four days, seeing adjacent towns; then to Dinant *via* the Meuse. Stay there a week or ten days, returning *via* Louvain and Alost. Third tour: Go straight from Southampton to St. Malo, spending the entire three weeks there and making all the numerous excursions around. Lodgings are non-existent on the Continent, in our sense of the word; you must go either to hotels or pensions. Write me again which of the three routes you like best, and exactly how much you can each afford to spend, and I will arrange for you as economically as possible.

RULES IN REGARD TO CORRESPONDENCE FOR THIS SECTION.—All questioners must use a pseudonym for publication, but the communication must also bear the writer's own name a... address as well, which will be regarded as confidential. All such communications to be addressed "Travel Correspondent, 28 Southampton Street, Strand." No charge will be made for inserting and answering questions in the inquiry column, and all will be answered in rotation as space permits. If an answer by letter is required, a stamped and addressed envelope must be enclosed, together with 2s. 6d., which fee will be devoted to the objects of "The Hospital" Convalescent Fund. Ten days must be allowed before an answer can be published.

### Notes and Queries.

**REGULATIONS.**

**The Editor is always willing to answer in this column, without any fee, all reasonable questions, as soon as possible.**

**But the following rules must be carefully observed.**

1. **Every communication must be accompanied by the name and address of the writer.**
2. **The question must always bear upon nursing, directly or indirectly.**

**If an answer is required by letter a fee of half-a-crown must be enclosed with the note containing the inquiry.**

*Scarlet Fever.*

(39) Will you kindly tell me if all authorities on scarlet fever are agreed that all genuine cases peel?—*H. E. M.*

The general opinion is that genuine cases peel, but some authorities make a reservation.

*Inspector of Midwives.*

(40) Kindly tell me what duties are expected of an inspector of midwives, also the salaries, and if special examinations have to be passed for the post. I have a certificate from Queen Charlotte's Hospital and Central Midwives Board. Also, is there any special age, and is general training necessary? I am considered an excellent midwife and well up in my work.—*Q. C. H.*

Write to the Central Midwives Board, 6 Suffolk Street, Pall Mall, S.W.

*Epileptic Lady.*

(41) Can you give me information as to where an incurable epileptic lady could be placed in home or institution for £20 a year? She is over 50 years of age, and can do crochet and fancy work.—*Nurse J.*

Write to the Secretary of the Liverpool Home for Epileptics, Maghull, near Liverpool. Or to the Secretary of the National Society for the Employment of Epileptics, 12 Buckingham Street, Strand, W.C., and ask him kindly to advise you.

(42) Please inform a reader of THE HOSPITAL of any institution or home for epileptics in the South of England suitable for a lady of 33 who can afford to pay about £1 a week.—*Parbury.*

See answer to *Nurse J.* The Meath Home of Comfort, Westbrook, Godalming, receives ladies up to 35 years of age at £1 1s. a week. Or write to the St. Luke's Home for Epileptic Churchwomen, 36 Parkwood Road, Bournemouth.

*Nursing in Egypt.*

(43) Will you kindly inform me if there is a hospital in Egypt where English trained nurses can apply for posts as sisters or charge nurses, and how I can get to know of vacancies?—*M. W.*

English nurses are employed at the Kasr-el-Ainy Hospital. But we fear your chance of success is small, as there are many candidates and few posts. You might hear of a vacancy by advertising.

*Chapped Hands.*

(44) Kindly tell me what is the best application to use on the hands to prevent and cure roughness arising from district work and the use of mercurial soap?—*E. K. W.*

Some people find lanoline rubbed in after washing is very good. Glycerine does not suit all skins. A very generally useful preparation is half castor oil and half toilet paraffin. If you use a very little after washing, and always rub a good quantity into your hands at night, afterwards putting on gloves with holes cut in the palms, you will probably find improvement.

*Training in the Midland Counties.*

(45) Are there any hospitals in Birmingham or the Midland Counties where I can apply for post as probationer, having had no previous experience? I should require a small salary.—*C. A. B.*

Consult "How to Become a Nurse," published by the Scientific Press, 28 Southampton Street, Strand, W.C.

*Dispensing.*

(46) I am anxious to learn dispensing. Can I do so at home? Kindly let me know the best and cheapest way.—*A. L.*

Write to Miss Buchanan, Gordon Hall, Gordon Square, W.C., who has arranged dispensing classes for women.

*Training in Provincial Hospitals.*

(47) I wish to train for three years in some general hospital with a view to entering the military nursing service. Can you tell me if such hospitals as Addenbrookes at Cambridge or the Portsmouth Hospital could grant a certificate that would be accepted by the War Office?—*Soldier.*

Yes, these hospitals are both recognised training schools.

*References.*

(48) I wish to receive training to be a nurse, and want to know if I would be expected to give references, and, if so, where could I obtain them?—*Primrose.*

Most hospitals now require references as to character and suitability for the life. You must surely know some friends who would answer for you. Your family doctor or the clergyman of your parish—if you know him—would probably act as referees.

*Partial Training.*

(49) I am a nurse with a little hospital experience; I have also attended lectures in home nursing. I now wish to take up some district work in or near Birmingham. Will you kindly tell me where to apply?—*F. P.*

We fear you have small chance of obtaining good work unless you train properly. Why not begin at once, and so qualify yourself for a good post?

*Electrolysis.*

(50) Will you kindly tell me in your paper the address of a professor of electrolysis or a beauty doctor in Leeds? I have a little mark I should like to have removed.—*B. L. Y.*

Consult your own doctor. We never give private recommendations.

*Asylum Training.*

(51) Can you tell me how I can get a nursing certificate? I have been over six years employed in a private asylum which does not go in for the Medico-Psychological certificate. Can I go in for it or any nursing certificate on my own account without an examination being held in the asylum where I am engaged?—*H. M.*

You can only obtain a nursing certificate by working for three years in a recognised training school. For particulars of the Medico-Pyschological Association's examination write to 11 Chandos Street, London, W.

*Poor-law Nurses and the Railway.*

(52) Can you inform me if Poor-law nurses are privileged to travel second class; also if they can travel first class with a third class ticket. If correct it would be to our advantage.—*K. W.*

You can, of course, travel second class if you pay to do so, but if you buy a third class ticket you must travel third class.

*Preliminary Study.*

(53) Will you tell me if there are any subjects I could study which would help me to become a nurse? I am 17½. Are there any hospitals which would take me when I am 18 years old?—*C. A. K.*

You are far too young. No general hospital will take you until you are 23. In the meantime you will be helping yourself for your future life by studying simple physiology. You might get from the Scientific Press, 28 Southampton Street, Strand, W.C., "Elementary Physiology for Nurses," price 2s. Then occupy yourself in learning simple cookery and housework.

*Army Nursing Service.*

(54) I wish to know if "Queen Alexandra's Imperial Military Nursing Service" and "The Army Nursing Service" are identical organisations? I am desirous of applying for the Army Nursing Service, and I shall feel grateful if you will tell me to whom I should write for particulars and form of application.—*I. B.*

Write for information to the Secretary, War Office, 68 Victoria Street, S.W.

*Free or Assisted Training in Midwifery.*

(55) Can you inform me where I could obtain training at a reduced rate?

Write to the Association for Promoting the Training and Supply of Midwives, Dacre House, Dean Farrar Street, Westminster, S.W.

*Lantern Slides.*

(56) Can you advise me as to the best way of obtaining lantern slides on home hygiene, etc?—*M. H.*

We do not reply by letter unless 2s. 6d. is sent. It is possible that you might find what you need by applying to the Sanitary Institute, Margaret Street, W.

*Fee for Private Nurse.*

(57) What is the usual fee for a private medical nurse?—*Inquirer.*

The usual fee is £2 2s. per week in London, and frequently less in the Provinces.

**Handbooks for Nurses.**

| | Post Free. |
|---|---|
| "A Handbook for Nurses." (Dr. J. K. Watson) ... | 5s. 4d. |
| "Nurses' Pronouncing Dictionary of Medical Terms" | 2s. 0d. |
| "Art of Massage." (Creighton Hale.) ... ... | 6s. 0d. |
| "Surgical Bandaging and Dressings." (Johnson Smith.) ... ... ... ... ... ... ... | 2s. 0d. |
| "Hints on Tropical Fevers." (Sister Pollard.) ... | 1s. 8d. |

Of all booksellers or of The Scientific Press, Limited, 28 & 29 Southampton Street, Strand, London, W.C.

### For Reading to the Sick.

### WAITING IN FAITH.

All my life I still have found,
 And I will forget it never,
Every sorrow hath its bound,
 And no cross endures for ever.
After all the winter's snows
 Comes sweet summer back again;
Patient souls ne'er wait in vain,
 Joy is given for all their woes.
All things else have but their day,
God's love only lasts for aye.

*Lyra Germanica.*

Our blessed Lord bearing His Passion, in the weakness of His sinless human nature, felt, what we so often feel, a sense of desolation; felt Himself to be, what He never could be, what no man ever can be in this life—forsaken by God.

When we see the sun set, and darkness comes on, we accept the night without alarm, because we know that the sun will rise again the next morning. How do we know this? Chiefly by the experience of the past. It has happened so many times that we have come to consider it a practical certainty, and call it—or miscall it—a law of Nature. But the day will come when the sun will not rise again. We do not know how near we are to that day, the day of the Second Advent of our Lord.

But while the time will come when day and night, summer and winter, shall be no more, the time will never come when God will forsake any one of His children. We may forsake Him and refuse to have Him for our God, for our Father. And He may allow us to persevere in our wilful choice, for He has given us the gift of free will, and will not override or take from us that gift which He has bestowed upon us. But of this we may be certain—He will never forsake us.

So in times of despondency and darkness, when we cannot see our way, and no ray of hope lightens our path, let us reflect on this promise. It is impossible that God should forsake us, as impossible as that the sun, around which this earth constantly moves, can forsake the earth.—*A. G. Mortimer, D.D.*

"There remaineth therefore a rest for the people of God." Not here—not here, but in that Paradise of pure delights, where I have gone to prepare a place for those that love Me. . . . Come unto Me, wounded and weeping, homeless and joyless, outcast and alone—come in tears, which none will wipe away—come in pain, which no man will relieve—come in agony, which all shall pass unheeding, and I—I will give thee rest.—*The Divine Master.*

Thou art with me, O my Father,
 In the changing scenes of life,
In loneliness of spirit,
 And in weariness of strife.
My sufferings, my comfortings,
 Alternate at Thy will;
I trust Thee, O my Father,
 I trust Thee, and am still.

*The Dove on the Cross.*

## Figures and Tables

**Fig. 6. f1:**
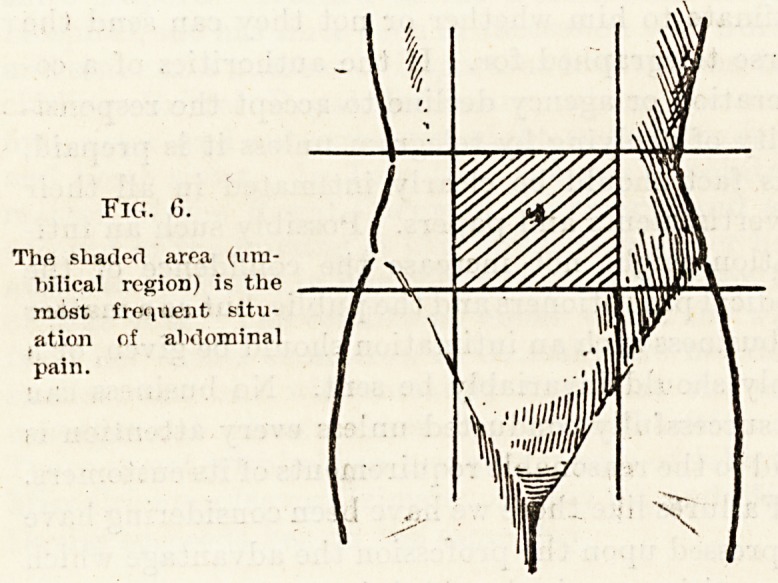


**Fig. 7. f2:**